# Host Specificity of the Bioherbicidal Fungal Strain *Paramyrothecium eichhorniae* TBRC10637 for Control of Water Hyacinth

**DOI:** 10.3390/biology14020199

**Published:** 2025-02-14

**Authors:** Tanyapon Siriphan, Arm Unartngam, Wachiraya Imsabai, Piyangkun Lueangjaroenkit, Chatchai Kosawang, Hans Jørgen Lyngs Jørgensen, Jintana Unartngam

**Affiliations:** 1 Department of Plant Pathology, Faculty of Agriculture at Kamphaeng Saen, Kasetsart University, Nakhon Pathom 73140, Thailand; tanyapon.si@ku.th (T.S.); chko@ign.ku.dk (C.K.); 2 Department of Science, Faculty of Liberal Arts and Science, Kasetsart University, Kamphaeng Saen Campus, Nakhon Pathom 73140, Thailand; faasarmu@ku.ac.th; 3 Department of Horticulture, Faculty of Agriculture at Kamphaeng Saen, Kasetsart University, Nakhon Pathom 73140, Thailand; wachiraya.i@ku.th; 4 Department of Microbiology, Faculty of Science, Kasetsart University, Bangkok 10900, Thailand; piyangkun.lu@ku.th; 5 Department of Geosciences and Natural Resource Management, University of Copenhagen, Rolighedsvej 23, 1958 Frederiksberg, Denmark; 6 Department of Plant and Environmental Sciences and Copenhagen Plant Science Centre, University of Copenhagen, Thorvaldsenvej 40, 1871 Frederiksberg, Denmark

**Keywords:** *Paramyrothecium eichhorniae* TBRC10637, water hyacinth, host specificity, bioherbicide

## Abstract

*Paramyrothecium eichhorniae* TBRC10637 has been reported as a potential biocontrol agent of water hyacinth (*Eichhornia crassipes*) in Thailand. Despite its great potential, it remained unclear whether the strain may cause disease in other plant species, especially those sharing the same niche as water hyacinth. Here, we examined the strain for its specificity and pathogenicity on 55 plant species from 26 families ranging from crop plants to aquatic weeds. We showed that, except for water hyacinth, *P. eichhorniae* TBRC10637 did not cause leaf spot or leaf blight or on any of the tested plants. Scanning electron microscopy of spores inoculated on eight plant species, including economically important plants such as maize (*Zea mays*) and chilli (*Capsicum annuum*) at 0, 24, 48, and 72 h after inoculation, showed no spore germination, except on water hyacinth. Inoculation with spore-free culture washing led to blight symptoms on leaves of water hyacinth 72 h after inoculation, suggesting that enzymes and secondary metabolites may be involved in causing the blight symptoms. Our results confirmed high specificity of *P. eichhorniae* TBRC10637 towards water hyacinth, paving the way to control the spread of water hyacinth effectively.

## Simple Summary

Water hyacinth is an invasive aquatic weed found in almost all continents across the globe. The plant has a rapid reproduction, making control of the highly invasive species a challenge. The use of bioherbicides to suppress water hyacinth is one of the most environmentally friendly control options. However, the majority of bioherbicides are fungal plant pathogens with a wide host range. In this study, we tested the biological control agent *Paramyrothecium eichhorniae* TBRC10637 on 55 plant species from 26 families and showed that it attacks only water hyacinth. Further investigation using light and scanning electron microscopy showed that TBRC10637 spores did not germinate on other plants. We also found that the strain produces toxic metabolites, which caused disease symptoms only on water hyacinth. The toxic metabolites are water-soluble, offering a new way to effectively control water hyacinth.

## 1. Introduction

Water hyacinth (*Eichhornia crassipes*, syn. *Pontederia crassipes*) is an aquatic weed in the family Pontederiaceae originating from the Amazon Basin in South America. It is considered an invasive species, causing social, economic, and environmental problems globally due to its high adaptability and fast reproduction [1,2,3]. Several methods have been proposed to control this aquatic weed from water resources. Among those are mechanical approaches, which involve the use of heavy machinery, chemical approaches, which rely on chemical herbicides and biological approaches, which use natural enemies, such as pests and pathogens [1,4]. Mechanical approaches are difficult to implement due the high-water content in the plants and the need for operating heavy machinery, while the use of chemical approaches has adverse effects on water quality and aquatic lives. The use of bioherbicides is considered a better option for controlling water hyacinth due to its low operational cost and environmentally friendliness [2,5].

Bioherbicides were first developed after the second world war, when various phytopathogenic organisms, including fungi, bacteria, and viruses, were tested [6]. Currently, only a few bioherbicides are commercially registered and available, the majority of which are plant pathogenic fungi to control agricultural weeds [7]. Interestingly, no bioherbicides are available for water hyacinth when considering the economic and ecological losses the species causes. Around 60 fungal species are reported to cause disease in water hyacinth, and of these, only 10 species can cause the aquatic plant to succumb [8]. These species are *Acremonium zonatum*, *Alternaria alternata*, *A. eichhorniae*, *Bipolaris* spp., *Cercospora piaropi*, *Fusarium chlamydosporum*, *Helminthosporium* spp., *Myrothecium roridum*, *Rhizoctonia solani*, and *Uredo eichhorniae*. Among these, *A. eichhorniae*, *A. zonatum*, *C. piaropi*, *R. solani*, *A. alternata*, and *M. roridum* have gained attention because of their potential for development as mycoherbicides [8,9]. In Thailand, *Myrothecium roridum* was first isolated and identified in 1974 as the causal agent of leaf blight on water hyacinth [10]. During 2013–2016, an attempt to find an effective strain of *M. roridum* to control water hyacinth in Thailand was made, and *M. roridum* KKFC448 was identified as a very potent candidate [4,11,12,13]. Since then, it has been used to suppress water hyacinth outbreaks in various water reservoirs across the country [12].

Until recently, *Myrothecium* was a cryptic genus and contained more than 30 species [14]. The use of multi-locus phylogenetic analysis has revised the genus *Myrothecium*, and some of the species have been assigned to the newly established genus *Paramyrothecium* [15]. Among these was *Myrothecium roridum*, which was renamed as *Paramyrothecium roridum* [15]. Due to the renaming, the taxonomical status of *M. roridum* KKFC448 was reanalysed using multi-locus phylogenetic analysis, and a new species, *Paramyrothecium eichhorniae*, was established. Therefore, *M. roridum* KKFC448 is now a type species and deposited at the Thailand Bioresource Research Center (TBRC) as *Paramyrothecium eichhorniae* TBRC10637 [16]. *P. eichhorniae* TBRC10637 has been widely tested as a bioherbicide. Since it is a potent pathogen of water hyacinth, the strain is certified and will soon be commercialised.

Currently, five species of *Paramyrothecium* have been isolated from 16 hosts in Thailand, i.e., *P. eichhorniae*, *P. vignicola*, *P. folliicola*, *P. breviseta*, and *P. amorphophalli* [16,17,18]. Withee et al. [18] recently reported five new hosts of *P. eichhorniae* from four families, i.e., paracress (*Acmella oleracea*), Dutchman’s pipe (*Aristolochia ringens*), Indian trumpet tree (*Oroxylum indicum*), winged bean (*Psophocarpus tetragonolobus*), and butterfly pea (*Centrosema* sp.). Although previous studies have suggested that *P. eichhorniae* TBRC10637 is specific to plants in the family Pontederiaceae [12,16], the fact that *P. eichhorniae* is a sister species of *Paramyrothecium foliicola*, a potent phytopathogen with a broad host range, raises concerns over the use of *P. eichhorniae* as a bioherbicide [12,15]. Therefore, it is important to examine the host specificity of *P. eichhorniae* TBRC10637 to ensure that it does not present unwanted side effects on other plant species. Accordingly, the objectives of this study were to evaluate the pathogenicity and host range of *P. eichhorniae* TBRC10637.

## 2. Materials and Methods

### 2.1. Host Range Determination

Fifty-five species from 26 families of native and economic plants were used to determine the host specificity of *P. eichhorniae* TBRC10637, which was isolated from water hyacinth from Chiang Mai, the northern province of Thailand [12] (Table 1). Plants were either grown in the greenhouse or purchased from plant nurseries. Host range determination was conducted in the greenhouse at Kasetsart University, Kamphaeng Saen Campus, Nakhon Pathom, Thailand. TBRC10637 spore suspensions and spray inoculations were used for the infection assay. The spore suspensions were prepared from 2-week-old potato dextrose agar cultures (Difco, Becton, Dickinson and Company, Bangkok, Thailand). The Petri dishes were flooded with 15 mL autoclaved distilled water, and a sterile L-shaped glass rod was used to scrape the surface of the culture medium. The spore suspension was filtered to remove mycelium using a fine metal mesh with a pore size of 1 mm and the spore suspension adjusted to 10^8^ spores/mL before spraying on to the plants. After spraying, the plants were covered with plastic bags to maintain high humidity and to promote infection. The plastic bags were removed after 48 h, and the plants were maintained in the greenhouse. Experiments took place during May–June. The relative humidity and temperature in the greenhouse were 80–90% and 25–32 °C, respectively, throughout the experiments. The pathogenicity of TBRC10637 was evaluated at 14 days after inoculation (DAI) [12].

### 2.2. Scanning Electron Microscopy of Infection Processes

To understand the infection biology of TBRC10637 better, eight plant species were selected for further investigation using scanning electron microscopy. Inoculated leaf samples were collected at 0, 24, 48, and 72 h after inoculation (HAI). The samples were cut into pieces of approximately 1 × 1 cm and fixed overnight with 2% (*v*/*v*) glutaraldehyde in 50 mM phosphate buffer (pH 7.2) as described in [19]. The fixed samples were washed in the same buffer three times, dehydrated in a graded series of ethanol (10, 20, 30, 40, 50, 60, 70, 80, 90, and 100%, *v*/*v*) at 1 h intervals, and dried using critical point drying [20] using a CPD750 critical point drier (Quorum Technology, East Sussex, UK). The dried samples were sputter-coated with gold (Polaron Range SC7620 Mini Sputter Coater, Quorum Technology, Kent, UK), and observations were made with a FEI Quanta 450 Scanning Electron Microscope (FEI Technology Inc., Hillsboro, OR, USA).

### 2.3. Effects of Spore-Free Culture Washing on Plant Leaves

TBRC10637 was inoculated into 15, 500 mL Erlenmeyer flasks containing 150 g boiled paddy rice (cv. KDML105) each, and the flasks were incubated at 26–28 °C for 14 days. The boiled paddy rice from the flasks were combined and washed with one litre autoclaved distilled water. The spores were removed from the culture washing by filtering through five layers of gauze, Whatman papers (no. 1 and no. 5), and finally a Whatman mixed cellulose ester WME filter disc with a pore size of 0.45 μm (Cytiva, Tokyo, Japan), following previously used methods [12].

In addition to the spray inoculation of spore-free culture washing on water hyacinth, the effects of the washing were tested on the eight species previously used for SEM studies (Table 1). Thus, sterile paper discs (1.8 cm in diameter) were saturated with the spore-free culture washing, and three discs were placed on the leaves of selected plants, i.e., water hyacinth (*Eichhornia crassipes*), yard-long bean (*Vigna unguiculata* ssp. *sesquipedalis*), heartleaf pickerel (*Pontederia cordata*), maize (*Zea mays*), cucumber (*Cucumis sativus*), giant taro (*Alocasia macrorrhizos*), chilli (*Capsicum annuum*), and Chinese kale (*Brassica oleracea* var. *alboglabra*) under greenhouse conditions. Symptoms were recorded visually as absence or presence of the symptoms at 1, 2, and 3 DAI. Disks saturated with sterile distilled water were used as controls.

### 2.4. Light Microscopy of Leaves Sprayed with Spore-Free Culture Washing

To evaluate structural damages induced by the spore-free culture washing, the washing was sprayed on water hyacinth leaves and collected at 0, 6, 12, 24, 36, 48, 60, 72, 84, 96, 168, and 240 h after treatment for light microscopy. The samples were prepared by cutting pieces (ca. 1.5 × 0.5 cm) and fixing them in 50% (*v*/*v*) FAA (10 mL formaldehyde 40% (*v*/*v*), 5 mL glacial acetic acid, 50 mL 95% (*v*/*v*) ethanol, 35 mL distilled water) for at least 48 h. The samples were washed with 50% (*v*/*v*) ethanol three times at 3–4 h intervals and were dehydrated in solutions of increasing concentrations of tertiary butyl alcohol from 50 to 100% (*v*/*v*). The dehydrated samples were embedded in paraffin (Gurr, VWR International Ltd., Lutterworth, UK), sectioned at 20 μm using a rotary microtome (Artisan Technology Group, Zevenhuizen, The Netherlands), and mounted on glass slides using Permount (ThermoFischer Scientific, Bangkok, Thailand) as an adhesive [21].

The paraffin was dissolved in xylene, and the slides were passed through a decreasing series of ethanol (95, 70, 50, and 30%, *v*/*v*). To enhance visualisation, the samples were stained with safranin O and fast green before rinsing them with distilled water and immersing them in an increasing series of ethanol concentrations (30, 50, 70, and 95%, *v*/*v*). The slides were then immersed in xylene for at least 6 h, dried, and mounted with Permount. The samples were examined using an Olympus CX31 light microscope (Olympus, Tokyo, Japan).

### 2.5. Phylogenetic Analysis

The phylogenetic analysis was carried out using concatenated nucleotide sequences of ITS rDNA, *tub2*, *cmdA*, and *rpb2*. Nucleotide sequences of these genes in *Paramyrothecium eichhorniae* isolates, including TBRC10637 and KKFC474, as well as other *Paramyrothecium* species and other fungal genera, were obtained from GenBank (NCBI) (Table 2). Multiple alignment was performed using ClustalW [22]. The aligned dataset was subject to phylogenetic analyses with maximum likelihood (ML) and Bayesian inference. The ML analysis was carried out in MEGA version 11 [23] with Tamura 3-parameter (TN92) as the best substitution model, Has Invariant Sites (I) as rates among sites, and 1000 bootstrap replications. The Bayesian inference was analysed using a MrBayes v.3.2.6 [24] plug-in of Geneious Prime R2024.0.7 (Biomatters, Auckland, New Zealand). The model K80+I+G4 determined by ModelTest-NG v0.1.7 [25] was used as the most suitable model for phylogenetic tree construction using 1,500,000 generations, using the first 5000 generations as burn-in.

## 3. Results

### 3.1. Host Range Determination

**Figure 1:**
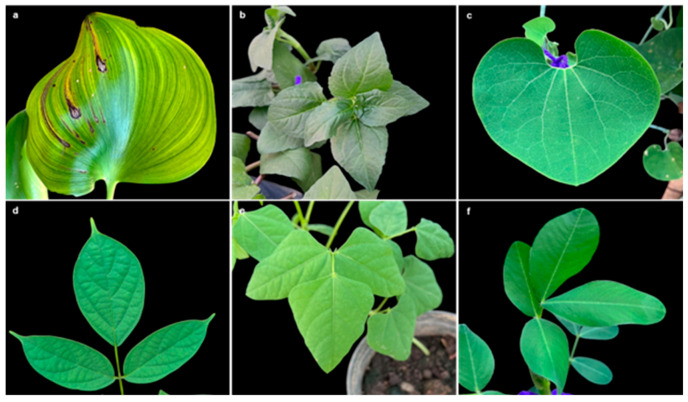
Appearances of leaves inoculated with *P. eichhorniae* TBRC10637 at 14 DAI: (**a**) water hyacinth (*Eichhornia crassipes*), (**b**) paracress (*Acmella oleracea*), (**c**) Dutchman’s pipe (*Aristolochia ringens*), (**d**) Indian trumpet tree (*Oroxylum indicum*), (**e**) winged bean (*Psophocarpus tetragonolobus*), and (**f**) butterfly pea (*Centrosema* sp.).

In general, inoculation of water hyacinth (Eichhornia crassipes) with *Paramyrothecium eichhorniae* TBRC10637 showed the typical symptoms with brown to dark brown spots, which developed into blight covering extended parts of the leaves within 2 weeks (Figure 1). While the screening for the host range of TBRC10637 showed the clear blight symptom on water hyacinth in this study, no disease symptoms were recorded on the other test plants from 26 families, including Monochoria hastata and Pontederia cordata, which belong to the same family as *E. crassipes* (Table 1). Symptoms developed on *E. crassipes* infected under greenhouse conditions were similar to the symptoms observed under natural conditions.

### 3.2. Scanning Electron Microscopy of Infection Processes

**Figure 2:**
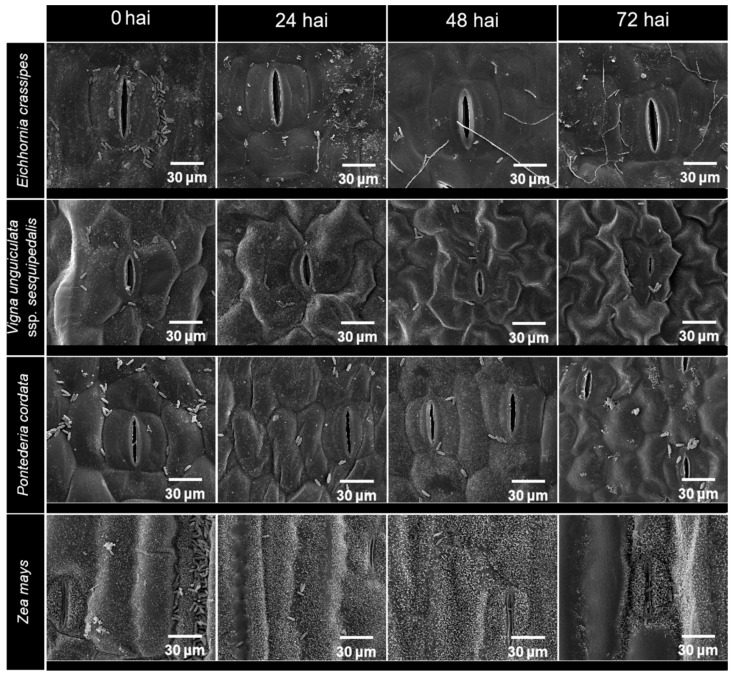
Scanning electron micrographs of germination of *Paramyrothecium eichhorniae* TBRC10637 spores on leaves of water hyacinth (*Eichhornia crassipes*) and non-host plants, i.e., yard-long bean (*Vigna unguiculata* ssp. *sesquipedalis*), heartleaf pickerel (*Pontederia cordata*), and maize (*Zea mays*) at 0, 24, 48, and 72 h after inoculation (HAI).

The SEM showed contrasting behaviours of the inoculated TBRC10637 spores on different host plants (Table 1). The spores inoculated on water hyacinth started germinating at 24 HAI, and almost all spores germinated by 48 HAI. On the contrary, spores inoculated on the other seven plant species from seven families with both close and distant genetic distance to water hyacinth failed to germinate (Figure 2). Neither germinated spores nor mycelial growth were found on the inoculated leaves of other tested plant species even after 72 h (Table 1, Figure S1).

### 3.3. Effects of Spore-Free Culture Washing on Plant Leaves

**Figure 3:**
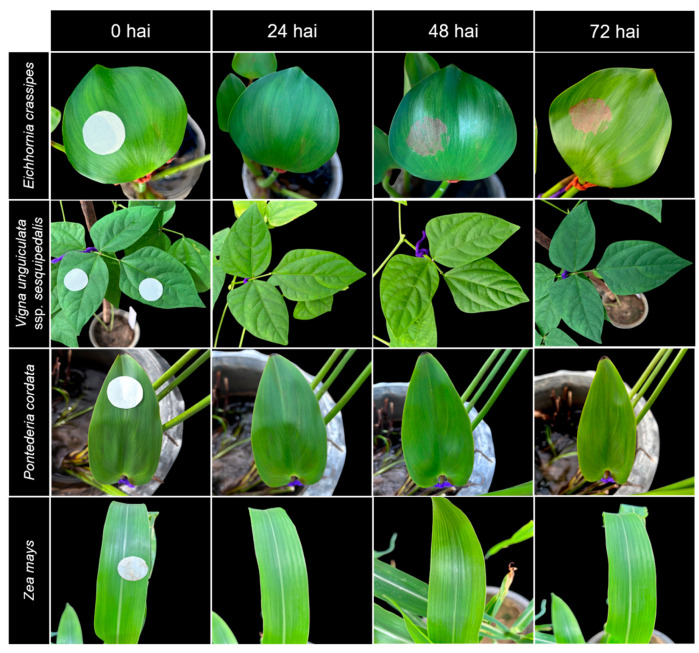
Appearance of symptoms after treatment with paper discs saturated with spore-free culture washing at 0, 24, 48, and 72 h after inoculation (HAI) on water hyacinth (*Eichhornia crassipes*) and non-host leaves, i.e., yard-long bean (*Vigna unguiculata* ssp. *sesquipedalis*), heartleaf pickerel (*Pontederia cordata*), and maize (*Zea mays*).

To learn more about how the infection of TBRC10637 takes place, leaves were treated with spore-free culture washing. The washing applied on water hyacinth caused blight and necrosis at 24 HAI, and the symptoms were fully developed at 72 HAI, when the infected leaves dried out. It was surprising that the leaf blight symptoms and dryness developed even faster than when using spore suspensions (Figure 3). This suggests an involvement of secondary metabolites in causing the symptoms and indicates the potential of using the spore-free washing as a bioherbicide.

Paper discs saturated with the spore-free culture washing were also applied on leaves of eight plant species previously used for SEM to examine effects of metabolites on other plant species (Table 1, Figure 3, Figure S2). Symptoms on leaves of water hyacinth started as drying at the point of infection, followed by subsequent expansion (blighting). None of the symptoms were seen on the other test plants (Figure 3). This provides evidence that the metabolites produced by TBRC10637 are specific to water hyacinth.

### 3.4. Light Microscopy of Leaves Sprayed with Spore-Free Culture Washing

**Figure 4:**
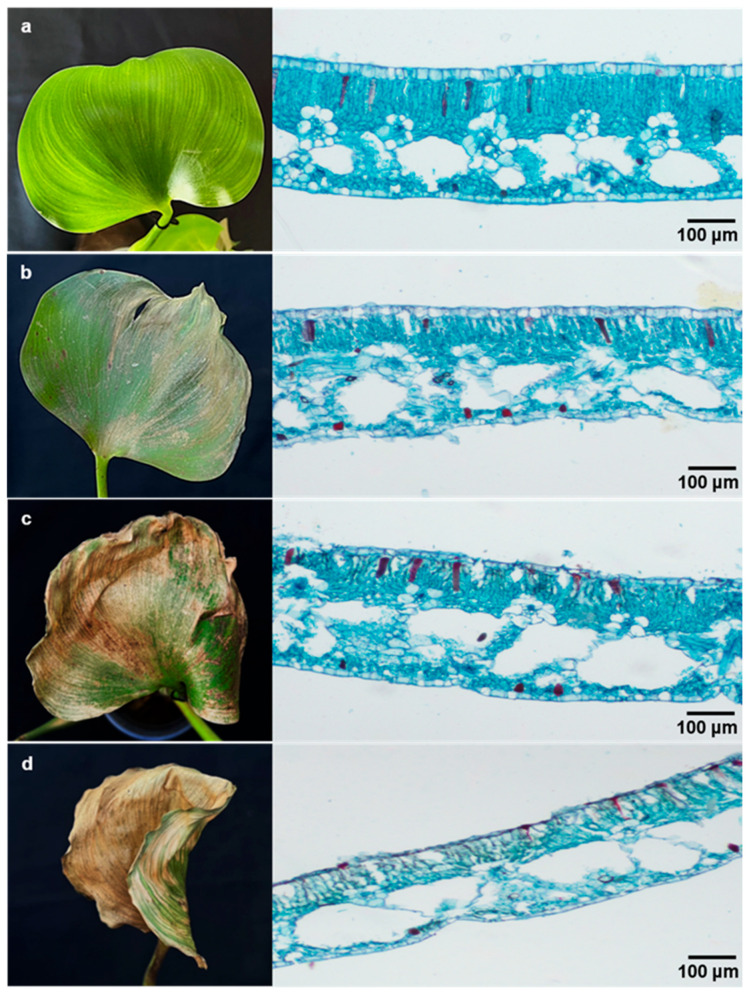
Microscopic structure of water hyacinth leaf cells after treatment with spore-free culture washing at (**a**) 0, (**b**) 24, (**c**) 48, and (**d**) 72 h after inoculation.

Light microscopy was used to examine the symptoms induced by the metabolites of TBRC10637. A healthy leaf of water hyacinth is composed mainly of densely arranged palisade cells vertically under the adaxial epidermis, whereas the epidermis is made of a single layer of rectangular cells (Figure 4a). On leaves sprayed with the spore-free culture washing, the upper epidermis cells turned brown, showing signs of distortion and cell leakage, and the upper palisade cells started collapsing (Figure 4b). After 48 h, the damage started enlarging and expanding towards the lower epidermal cells, and, especially, the palisade cells started disintegrating (Figure 4c). The damaged cells and areas expanded significantly at 72 HAI, when the upper epidermal cells were disintegrated, and became sunken and completely misshaped. Likewise, the upper palisade cells were completely destroyed, resulting in a more shrivelled appearance and cell loss in some areas (Figure 4d). Since the spore-free washing was used, the observed symptoms must be due to the effects of secondary metabolites.

### 3.5. Phylogenetic Analysis

**Figure 5:**
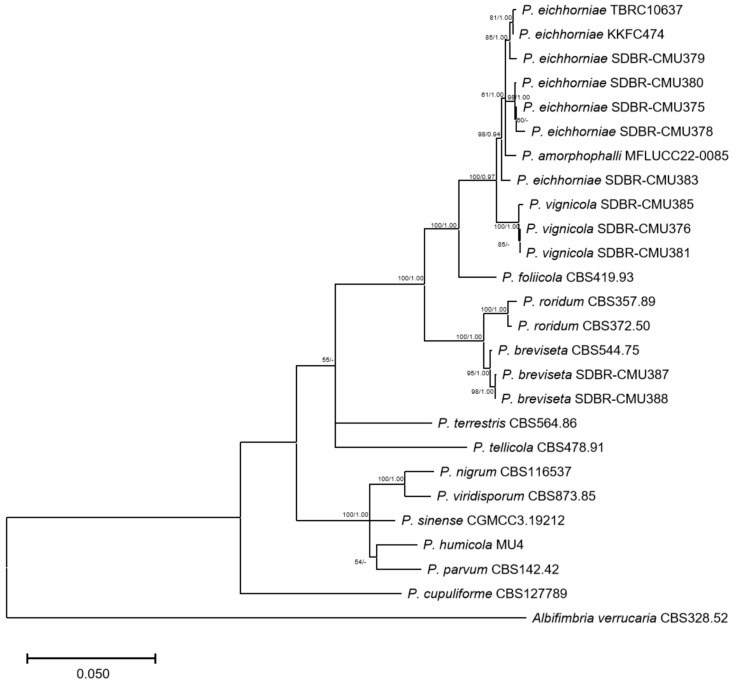
Phylogenetic relationships of *Paramyrothecium* spp. from concatenated nucleotide sequences of ITS rDNA, *tub2*, *cmdA*, and *rpb*2 analyses. *Paramyrothecium eichhorniae* TBRC10637 was placed. The bootstrap values (1000 replications) and Bayesian posterior probabilities over 50% and 0.90 were shown on the left of the nodes, respectively.

Multi-loci phylogenetic analysis placed TBRC10637 together with the other *P. eichhorniae* isolates regardless of the methods used for phylogenetic tree construction (Figure 5). Nonetheless, *P. eichhorniae* TBRC10637 formed a small branch along with its sister isolate KKFC474, branching out from the rest of the *P. eichhorniae* isolates. It is noteworthy that both TBRC10637 and KKFC474 originated from water hyacinth, whereas the other *P. eichhorniae* isolates were derived from other host plants, including *Acmella oleracea* (paracress), Aristolochia ringens (Dutchman’s pipe), *Oroxylum indicium* (Indian trumpet tree), *Centrosema* sp. (butterfly pea), and *Psophocarpus tetragonolobus* (winged bean). Taken altogether, these sets of evidence did not only validate the taxonomic status of TBRC10637 as *P. eichhorniae* but also supported the hypothesis that strains of *Paramyrothecium eichhorniae* may be subdivided according to their host plants, and this specificity would indicate that isolate TBRC10637 belongs to a forma specialis attacking water hyacinth, i.e., TBRC10637 is *P. eichhorniae* f.sp. *P. eichhorniae* (Table 1).

## 4. Discussion

The *Paramyrothecium eichhorniae* strain TBRC10637 was proposed as a new species in the genus *Paramyrothecium* [16]. Recently, five new species of *Paramyrothecium* were isolated from new hosts [18]. *P. eichhorniae* is a sister species of *P. foliicola* [16], which is a leaf, stem, and fruit pathogen of many plant species [26,27,28]. This has raised concerns over the use of the strain as a bioherbicide, as it may cross-infect other potential host plants and cause ecological/economic losses [29,30]. To address the potential pathogenicity of TBRC10637, we paid special attention to cash crops in this study and screened its host range by inoculating a total of 55 plant species from 26 families. The fact that no symptoms were observed on any of the tested plants, including *Monochoria hastata* and *Pontederia cordata*, which belong to the same family as water hyacinth, suggests differences in both pathogenicity and host specificity within *P. eichhorniae*.

Spore germination of fungal pathogens is a crucial process, as it determines the survival of spores and whether disease establishment will be successful. In many fungal pathogen species, spores generally germinate shortly after they obtain contact with their plant hosts. For example, spores of the rice blast fungus Pyricularia oryzae and the anthracnose fungus *Colletotrichum graminicola* start germinating as fast as two and six hours after inoculation on their hosts, respectively [31,32]. The fact that the spores of TBRC10637 did not germinate on other plant species provides evidence supporting the hypothesis that the pathogenicity of TBRC10637 is specific to water hyacinth. Additionally, several factors are known to influence spore germination, including temperature, light, moisture, and plant secondary metabolites [33]. Chaky et al. [34] showed that surface hydrophobicity and rigidity affect germination in Colletotrichum graminicola. Whether the same factors modulate germination of TBRC10637 remain to be determined. Piyaboon et al. [12] suggested that *P. stratiotes* can be a host of TBRC10637. However, we did not obtain the same result in this study and failed to re-isolate the fungus after inoculation. It is therefore unlikely that *P. stratiotes* is a potential host of TBRC10637.

Cell-free culture washing from *Paramyrothecium eichhorniae* was able to cause symptoms on water hyacinth, but not in several other plant species tested. Previous discoveries in other fungal–plant pathosystems have underlined the functions of host-specific toxins as determinants of compatibility in plant–fungal interactions [35,36,37]. Host-specific toxins have not been discovered in *P. eichhorniae* or even in the genus Paramyrothecium. However, species in the genus Myrothecium are known to produce diverse trichothecene metabolites, including roridin E, verrucarin, and mytoxin B [38,39,40]. Some trichothecenes are heat-stable [41,42], and some act as virulence factors [43,44]. Since the genus Paramyrothecium is derived from Myrothecium, it is likely that both genera share similar metabolic profiles. Further studies are therefore needed to determine the types of trichothecenes produced by and whether its host specificity is driven by those metabolites. 

We noticed the damaged cells of water hyacinth after treatment with the cell-free culture washing. Cell collapse and damages at subcellular level could suggest the involvement of cell-wall-degrading enzymes. *Paramyrothecium eichhorniae* has the ability to produce cell-wall-degrading enzymes (CWDEs), such as cellulolytic and xylanolytic enzymes [45,46]. Other studies found that *P. eichhorniae* TBRC10637 was able to produce five CWDEs, i.e., β-1,4-endoglucanase, β-1,4-exoglucanase, β-glucosidase, xylanase, and pectinase [12]. The reported enzymes could be important due to the fact that hemicellulose is a main content of water hyacinth cells together with cellulose, lignin, and little pectin and protein [47]. Of the five CWDEs, xylanase and pectinase were suspected to be the main responsible enzymes for the symptoms. However, Chaibang et al. [48] reported that xylanase alone was insufficient to cause the symptoms, indicating that the action of a cocktail of enzymes is required for the symptom development. Owing to the severe problem of water hyacinth, it should be determined whether low-molecular-weight CWDEs, secondary metabolites, or both are keys to the blight symptoms.

In many fungal–plant pathogenic species, strict pathogenic differentiation is observed. For example, 106 and 8 *formae speciales* were documented in *Fusarium oxysporum* and *Blumeria graminis*, respectively [49,50]. Thus, isolates from one *forma specialis* will attack certain plants, but not others, even if the plant species is generally attacked by the pathogen species. In some instances, previous *forma specialis* are turned into species, as has happened for *Blumeria graminis* f.sp. *hordei*, which is now known as *Blumeria hordei* [51]. Together with the phylogenetic analysis that branched TBRC10637 and its sister isolate off from the remaining *P. eichhorniae*, the high specificity for water hyacinth in this study provides evidence that the TBRC10637 strain of *P. eichhorniae* is potentially *P. eichhorniae* f. sp. *P. eichhorniae.*

## 5. Conclusions

*Paramyrothecium eichhorniae* TBRC10637 is a new potential biological control agent isolated from water hyacinth in Thailand. Its spores can germinate naturally on the host, but not on other tested plants, indicating its high specificity toward water hyacinth. The isolate also produces secondary metabolites, which can be secreted to the culture medium and cause leaf blight only on water hyacinth. The high specificity of TBRC10637 towards water hyacinth paves the way for an efficient control of this invasive aquatic weed worldwide.

## Figures and Tables

**Table 1:** Plant species included in tests of host specificity of *Paramyrothecium eichhorniae* TBRC10637 and their reaction (symptom expression) to spore suspensions and spore-free culture washing. Germination of spores studied by scanning electron microscopy is also indicated.

Plant Family	Scientific Name	Age at Inoculation ^a^	Symptoms ^b^	SEM ^c^	Spore-Free Culture Washing ^d^
Pontederiaceae	*Eichhornia crassipes* (host)	unknown	+	+	+
	*Monochoria hastata*	unknown	−	nt	nt
	*Pontederia cordata*	unknown	−	−	−
Araceae	*Alocasia macrorrhizos*	unknown	−	−	−
	*Colocasia esculenta*	unknown	−	nt	nt
	*Lemna minor*	unknown	−	nt	nt
Alismataceae	*Limnocharis flava*	unknown	−	nt	nt
Amaranthaceae	*Amaranthus albus*	30	−	nt	nt
Aristolochiaceae	*Aristolochia ringens*	unknown	−	nt	nt
Asteraceae	*Acmella oleracea*	unknown	−	nt	nt
	*Chromolaena odorata*	unknown	−	nt	nt
	*Lactuca sativa*	30	−	nt	nt
	*Lactuca sativa*	30	−	nt	nt
	*Lactuca sativa*	30	−	nt	nt
Basellaceae	*Basella alba*	30	−	nt	nt
Bignoniaceae	*Oroxylum indicum*	unknown	−	nt	nt
Brassicaceae	*Brassica chinensis* var. *chinensis*	30	−	nt	nt
	*Brassica rapa* susbp. *chinensis*	30	−	−	−
	*Brassica rapa* sp. *pekinensis*	30	−	nt	nt
	*Brassica oleracea* var. *alboglabra*	30	−	nt	nt
	*Eruca sativa*	30	−	nt	nt
Caricaceae	*Carica papaya*	30	−	nt	nt
Commelinaceae	*Commelina benghalensis*	unknown	−	nt	nt
	*Commelina diffusa*	unknown	−	nt	nt
Cucurbitaceae	*Citrullus lanatus*	30	−	nt	nt
	*Cucumis sativus*	30	−	−	−
	*Cucumis melo*	30	−	nt	nt
	*Cucurbita moschata*	30	−	nt	nt
Convolvulaceae	*Ipomoea aquatica*	30	−	nt	nt
Fabaceae	*Centrosema* sp.	30	−	nt	nt
	*Clitoria ternatea*	unknown	−	nt	nt
	*Glycine max*	30	−	nt	nt
	*Psophocarpus tetragonolobus*	30	−	−	−
	*Vigna radiata*	30	−	nt	nt
	*Vigna unguiculata*	30	−	nt	nt
	*Vigna unguiculata* ssp. *sesquipedalis*	30	−	−	−
Lamiaceae	*Ocimum × africanum*	30	−	nt	nt
	*Ocimum basilicum*	30	−	nt	nt
	*Ocimum gratissimum*	30	−	nt	nt
Malvaceae	*Abelmoschus esculentus*	30	−	nt	nt
Meliaceae	*Swietenia macrophylla*	unknown	−	nt	nt
Piperaceae	*Peperomia pellucida*	unknown	−	nt	nt
Plantaginaceae	*Plantago major*	unknown	−	nt	nt
Poaceae	*Oryza sativa*	30	−	nt	nt
	*Zea mays*	30	−	−	−
Polygonaceae	*Persicaria odorata*	unknown	−	nt	nt
Rosaceae	*Fragaria x ananassa*	30	−	nt	nt
Rubiaceae	*Coffea arabica*	30	−	nt	nt
	*Coffea canephora*	30	−	nt	nt
Rutaceae	*Citrus hystrix*	30	−	nt	nt
Solanaceae	*Capsicum annuum*	30	−	−	−
	*Nicotiana tabacum*	30	−	nt	nt
	*Solanum lycopersicum*	30	−	nt	nt
	*Solanum melongena*	30	−	nt	nt
Verbenaceae	*Tectona grandis*	unknown	−	nt	nt

^a^, Plants grown in greenhouse were inoculated when 30 days old. Unknown denotes that plants were purchased from plant nurseries, and therefore, age is unknown. ^b^ +, leaf blight observed; −, no leaf blight/spot observed. ^c^ +, germinated; −, not germinated; nt, not tested. ^d^ +, leaf blight observed; −, no leaf blight/spot observed; nt, not tested.

**Table 2:** Taxa with corresponding GenBank accession numbers used in the phylogenetic analysis.

Taxa	Strain	Substrate	Country	GenBank Accession Number			
				ITS	*cmdA*	*tub2*	*rpb2*
*Albifimbria verrucaria*	CBS328.52 ^T^	baled cotton	USA	KU845893	KU845875	KU845969	KU845931
*Paramyrothecium amorphophalli*	MFLUCC22-0085 ^T^	*Amorphophallus* sp.	Thailand	OP279643	OP434479	OP434480	OP434481
*P. breviseta*	CBS544.75 ^T^	unknown	India	KU846289	KU846262	KU846406	KU846351
*P. breviseta*	SDBR-CMU387	*Coffea arabica*	Thailand	MZ373251	OM810407	OM982450	ON033773
*P. breviseta*	SDBR-CMU388	*C. arabica*	Thailand	MZ373252	OM810408	OM982451	ON033774
*P. cupuliforme*	CBS127789 ^T^	soil	Namibia	KU846291	KU846264	KU846408	KU846353
*P. eichhorniae*	TBRC10637 ^T^	*Eichhornia crassipes*	Thailand	MT973996	MT975319	MT977540	MT975317
*P. eichhorniae*	KKFC474	*E. crassipes*	Thailand	MT973995	MT975318	MT975316	MT977541
*P. eichhorniae*	SDBR-CMU375	*Psophocarpus* sp.	Thailand	MZ373241	OM810411	ON033770	ON033781
*P. eichhorniae*	SDBR-CMU378	*Oroxylum indicum*	Thailand	MZ373246	OM810414	ON033772	ON033782
*P. eichhorniae*	SDBR-CMU379	*Spilanthes* sp	Thailand	MZ373247	OM810415	ON033768	ON033783
*P. eichhorniae*	SDBR-CMU38	*Centrosema* sp.	Thailand	MZ373250	OM810416	ON033784	ON033768
*P. eichhorniae*	SDBR-CMU383	*Aristolochia* sp.	Thailand	MZ373255	OM810418	ON033785	ON033769
*P. foliicola*	CBS419.93	air	Cuba	KU846293	KU846265	KU846410	KU846355
*P. humicola*	MU4	*Citrullus lanatus*	USA	MN227389	MN593629	MN398054	MN397959
*P. nigrum*	CBS116537 ^T^	soil	Spain	KU846296	KU846267	KU846413	KU846357
*P. parvum*	CBS142.42	dune sand	France	KU846297	KU846268	KU846414	KU846358
*P. roridum*	CBS357.89 ^T^	*Gardenia* sp.	Italy	KU846300	KU84627	KU846417	KU846361
*P. roridum*	CBS372.50	*Coffea* sp.	Colombia	KU846301	KU846271	KU846418	KU846362
*P. sinense*	CGMCC3.19212 ^T^	rhizosphere soils of *Poa* sp.	China	MH793296	MH885437	MH793313	MH818824
*P. tellicola*	CBS478.91 ^T^	soil	Türkiye	KU846302	KU846272	KU846419	KU846363
*P. terrestris*	CBS564.86 ^T^	soil	Türkiye	KU846303	KU846273	KU846420	KU846364
*P. vignicola*	SDBR-CMU376 ^T^	*Vigna* sp.	Thailand	MZ373242	OM810412	ON009015	ON033778
*P. vignicola*	SDBR-CMU381	*Commelina benghalensis*	Thailand	MZ373253	OM810417	ON009017	ON033780
*P. vignicola*	SDBR-CMU385	*Vigna* sp.	Thailand	MZ373257	OM810420	ON009018	ON033787

CBS: Westerdijk Fungal Biodiversity Institute, Utrecht, The Netherlands; CGMCC: China General Microbiological Culture Collection Center, Beijing, China; KKFC: Kasetsart Kamphaengsaen Fungal Collection, Thailand; SDBR-CMU: the Culture Collection of the Sustainable Development of Biological Resources Laboratory, Faculty of Science, Chiang Mai University, Chiang Mai, Thailand; TBRC: Thailand Bioresource Research Center, Thailand. ^T^ indicates type species.

## Data Availability

The original contribution presented in the study are included in the article/Supplementary Materials. Further enquiries can be directed to the corresponding authors.

## Supplementary Materials

The following supporting information can be downloaded at https://www.mdpi.com/article/10.3390/biology14020199/s1: Figure S1: Scanning electron microscopy of *P. eichhorniae* TBRC10637 on different non-host plants; Figure S2: Disease symptom on leaves of host and non-host plants after infection with *P. eichhorniae* TBRC10637.
