# Supplementary figures and images for: Host Specificity of the Bioherbicidal Fungal Strain Paramyrothecium eichhorniae TBRC10637 for Control of Water Hyacinth
Source: Biology (Basel). 2025 Feb 14;14(2):199. doi: 10.3390/biology14020199 (PMC11851953; doi:10.3390/biology14020199)

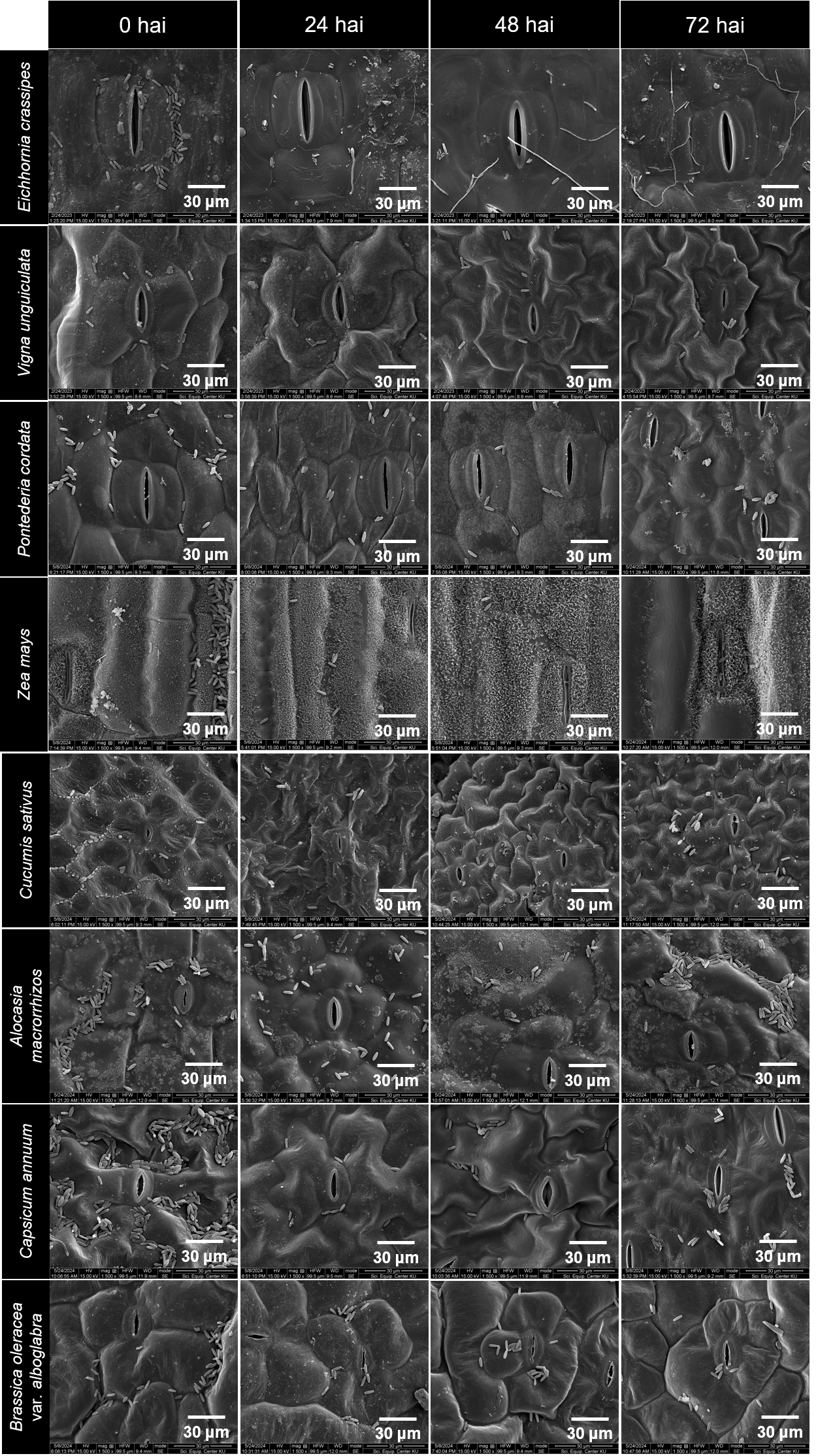

Supplement: Supplementary file 1 [file biology-14-00199-s001.zip › Figure S1.png]

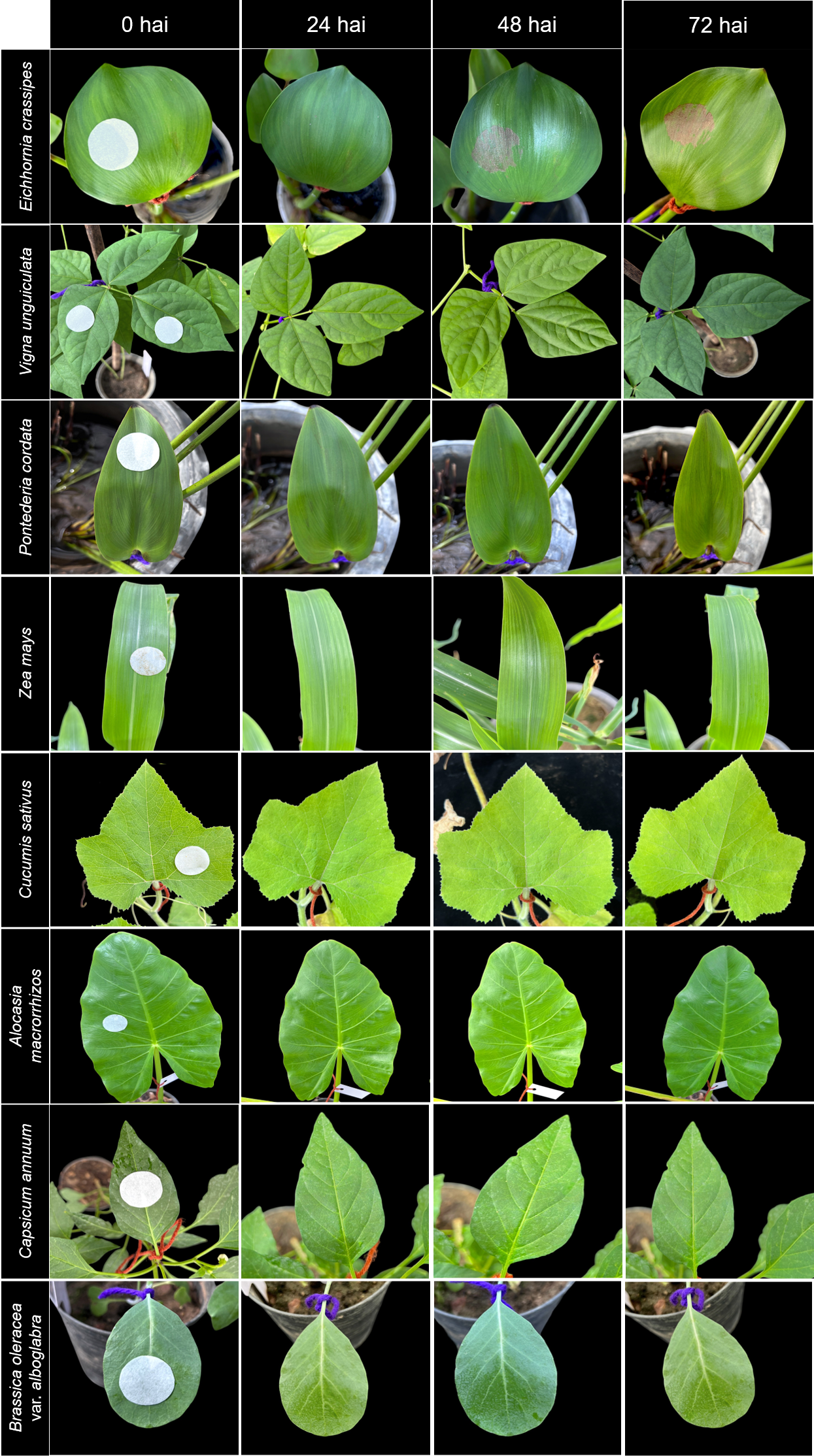

Supplement: Supplementary file 1 [file biology-14-00199-s001.zip › Figure S2.png]
